# General and Eating Disorder Specific Flexibility: Development and Validation of the Eating Disorder Flexibility Index (EDFLIX) Questionnaire

**DOI:** 10.3389/fpsyg.2019.00663

**Published:** 2019-04-02

**Authors:** Camilla Lindvall Dahlgren, Trine Wiig Hage, Joseph Arthur Wonderlich, Kristin Stedal

**Affiliations:** ^1^Regional Department for Eating Disorders, Division of Mental Health and Addiction, Oslo University Hospital, Oslo, Norway; ^2^Department of Psychology, George Mason University, Fairfax, VA, United States

**Keywords:** eating disorders, anorexia nervosa, bulimia nervosa, binge eating disorder, flexibility, cognition, self-report questionnaire

## Abstract

Findings from studies investigating cognitive flexibility in eating disorders (EDs) are inconsistent, and although neuropsychological tests are commonly used to measure these skills, they may not be particularly effective in predicting everyday functioning. Also, extant studies have largely focused on flexibility in anorexia nervosa (AN), with assessments targeting general rather than specific flexibility, and cognitive, rather than behavioral flexibility. Knowledge regarding ED specific flexibility and flexibility in bulimia nervosa (BN) and binge eating disorder (BED) is still scarce. The aim of this study was to develop and validate a novel measure assessing general and ED specific flexibility in a diagnostically diverse sample, and in healthy controls (HCs). A sample of 207 adult individuals with EDs (55% AN, 29% BN, 16% BED) and 288 HCs responded to an online, 51-item, pilot questionnaire on ED specific and general flexibility. In addition, participants completed the shift subscale from the Behavior Rating Inventory of Executive Function Adult version (BRIEF-A), and the Eating Disorder Diagnostic Scale (EDDS). A principal component analysis (PCA) in the clinical sample yielded a 36-item, three-factor solution capturing *general* flexibility, flexibility related to *food and exercise*, and flexibility concerning *body shape and weight*. Results showed that the measure had good to excellent internal consistency, and good convergent validity. A confirmatory factor analysis (CFA) using data from HCs revealed good fit indexes, supporting the original factor solution. A receiver operating characteristics analysis (ROC) demonstrated excellent accuracy in distinguishing scores from those with and without EDs. A cutoff score of 136 yielded the most balanced sensitivity and specificity. Significant differences in general and ED specific flexibility were found between individuals with and without EDs. Overall, HCs achieved the highest flexibility scores, followed by those with BED, BN, and AN. In sum this novel measure, the Eating Disorder Flexibility Index (EDFLIX) questionnaire, was found to be reliable and valid in the assessment of cognitive and behavioral flexibility, with results offering support for the conceptual distinction between general and ED related flexibility. The study also provides strong evidence for the discriminant validity of the EDFLIX with results revealing significant differences in flexibility in people with and without EDs. In addition, significant differences in flexibility also emerged when comparing diagnostic groups, indicating the utility of the assessment instrument for classification purposes.

## Introduction

Eating disorders (EDs) comprise a cluster of severe, often disabling, mental disorders characterized by abnormal eating patterns, purging behaviors and over-evaluation of shape and weight (Dahlgren and Wisting, 2016). The Diagnostic and Statistical Manual of Mental Disorders (DSM-5) (American Psychiatric Association, 2013) lists three main diagnostic ED categories; anorexia nervosa (AN), bulimia nervosa (BN), and binge eating disorder (BED). An additional diagnostic category labeled other specified feeding and eating disorders (OSFED) captures residual EDs failing to reach full-threshold criteria. Anorexia nervosa has the highest mortality rate of all mental disorders due to severe physical complications and suicide (Kask et al., 2016). The disorder is characterized by persistent efforts to lose weight despite being severely underweight, and an undue influence of body shape and weight upon self-evaluation. Concern regarding body image is also a core diagnostic criterion for BN. In addition, individuals with BN engage in frequent episodes of binge eating defined as eating, in a discrete period of time, an amount of food that is definitely larger than most people would eat during similar circumstances, whilst experiencing lack of control over eating during the episode. In BN, binge eating episodes are frequently followed by purging behaviors such as self-induced vomiting, misuse of laxatives and diuretics or excessive exercise. Binge eating disorder was first introduced as a formal ED diagnosis in the DSM-5 (American Psychiatric Association, 2013), and is like BN, characterized by recurrent binge eating episodes. Unlike BN however, binge eating is not followed by purging. According to a recent review from the United States, BED has the highest lifetime prevalence of all full-threshold ED diagnoses, approximately 3% (Brownley et al., 2016), whereas a review covering global prevalence estimates showed that AN and BN are less common with prevalence rates of 0.2 and 0.8% respectively (Qian et al., 2013). All three EDs are more common in females than in males, and are associated with substantial burden on physical and mental health (Dahlgren and Qvigstad, 2018).

The importance of cognitive flexibility as a fundamental aspect of health has gained widespread emphasis in recent years (Kashdan and Rottenberg, 2010; Rutten et al., 2013), also in the EDs field (Smith et al., 2018). Cognitive and behavioral flexibility is a key component of executive function, and relates to the ability to adapt and effectively shift thoughts and actions to the demands of the situation (Gioia et al., 2000). It has been suggested that executive dysfunction can explain some of the symptoms seen in patients with EDs, and specifically set-shifting deficits have been proposed being a salient neuropsychological phenomenon across both EDs and obesity (Wu et al., 2014). Set-shifting, a component of cognitive flexibility, has been proposed as a core feature of AN (Sultson et al., 2016), and some have even hypothesized that poor set-shifting could be an AN endophenotype (Tenconi et al., 2010; Talbot et al., 2015). Others have argued that the evidence base supporting set-shifting as AN endophenotype is insufficient, and that core biomarker criteria are missing (Micali and Dahlgren, 2016; Brown et al., 2018). Set-shifting has also been investigated in BN and BED where results have been mixed. Whereas some studies indicate that patients with BN and BED are on the other spectrum of the flexibility scale, i.e., that they display more difficulties with inhibitory control than flexibility (Svaldi et al., 2014; Manasse et al., 2015; Aloi et al., 2018), other studies report results similar to those in AN, i.e., enhanced inflexibility (Vall and Wade, 2015; Voon, 2015; Weider et al., 2015).

The construct of set-shifting in EDs is commonly used as a broad and encompassing term, not distinguishing between cognitive and behavioral shifting of responses (Zastrow et al., 2009). However, it has been proposed that inflexibility can be conceptualized as both impaired cognitive set-shifting, manifesting as rigid and direct approaches to rule changes, and as impaired behavioral response shifting – manifested as behaviors which are stereotyped and/or perseverative (Zastrow et al., 2009). The construct of cognitive-behavioral inflexibility has high face validity with clinical observations of patients with AN, as well as from parent reports (Dahlgren et al., 2014). The observed behaviors often reveal difficulties when plans are changed, with adapting to a different perspective or in changing strategies – all of which could indicate deficits in flexibility. Cognitive and behavioral inflexibility may manifest in aspects of the patients’ everyday life and lead to problems with general flexibility, but they may also manifest in terms of ED psychopathology, thereby resulting in problems with ED specific flexibility (Aloi et al., 2015; Dingemans et al., 2015). General inflexibility can manifest in rigid housekeeping or homework routines, but also in the social realm with interactions characterized by strict rules and regimes (Dahlgren and Stedal, 2017). Examples of ED specific inflexibility include strict rules around food preparation and eating, as well as ritualized weighing, body checking and exercise routines (Wu et al., 2014). Cognitive and behavioral inflexibility serves as a pervasive feature in individuals with EDs making therapeutic interventions challenging. Although face validity with cognitive validity is explored to a lesser degree for individuals with binge-spectrum EDs, difficulties with inhibitory control can make it more difficult to protect oneself against chaotic eating patterns, which may increase the likelihood of binge eating episodes in patients with BED and BN, or compensatory behaviors, like self-induced vomiting in patients with BN (Svaldi et al., 2010; Zakzanis et al., 2010). Moreover, poor set-shifting appears to be associated with longer duration of illness (DOI) and more severe ED rituals (Roberts et al., 2010). It is therefore of clinical interest to examine how these neurocognitive styles may help explain behaviors as well as serve as maintaining factors.

It is commonly assumed that the inflexible behaviors seen in patients with EDs, in particular patients with AN, are related to cognitive and behavioral flexibility deficits. However, the link between the often observed cognitive and behavioral rigidity, and results derived from neuropsychological, performance based assessments (Herbrich et al., 2018) remains elusive. Inconsistency between studies could be, in part, due to discrepancies in tasks used, but also due to heterogeneity in study samples, both in terms of age, diagnostic sub-groups and illness duration. Furthermore, it has been suggested that the traditional performance based tasks commonly used to assess cognitive flexibility (e.g., the Stroop, the Wisconsin Card Sorting Task or the Trail Making Test) in patients with EDs, are not optimal for this patient group as they were developed with the intent of assessing brain trauma and lesions, not psychiatric populations, and that they lack ecological validity and therefore are unable to capture the complexity of cognitions and behaviors in an interactive environment (Lounes et al., 2011; Stedal and Dahlgren, 2015). Neuropsychological testing is still considered to provide the most accurate profile of cognitive functioning. However, self-report questionnaires are cost-effective and easily administered, compared to neuropsychological testing. Self-report measures can also be useful in informing about patients’ perceived levels of cognitive-behavioral flexibility and to assess therapeutic changes in ED treatment. As such, they may be seen as valuable complimentary measures to neuropsychological testing (Lounes et al., 2011; Duarte et al., 2017). This has led to the development of several self-report questionnaires designed to assess cognitive flexibility, e.g., the Psychological Flexibility Questionnaire (PFQ) (Ben-Itzhak et al., 2014), the Cognitive Flexibility Scale (Martin and Rubin, 1995) and the Coping Flexibility Scale (Kato, 2012). To date, three self-report measures have been developed to assess flexibility in EDs; the Detail and Flexibility Questionnaire (DFlex) (Roberts et al., 2011), the Inflexible Eating Questionnaire (IEQ) (Duarte et al., 2017), and the Body Image Acceptance and Action Questionnaire (BI-AAQ) (Sandoz et al., 2013). The DFlex assesses cognitive rigidity and attention to detail in everyday situations, i.e., in a *general* context, but does not address topics or themes directly linked to EDs (e.g., food, eating, weight, or shape). Results from the DFlex have shown significant differences in flexibility when comparing individuals with lifetime EDs and HCs, and also in individuals with current and recovered AN. The IEQ and BI-AAQ on the other hand, both target cognitions and behaviors specific to EDs, but each measure focuses on one distinct target area, eating rules in the IEQ, and body image in the BI-AAQ. Moreover, the BI-AAQ and the IEQ primarily targets psychological rather than cognitive flexibility. Also, the IEQ has not been validated in a clinical population.

To date, no studies have broadly assessed ED specific cognitive-behavioral flexibility, i.e., targeted multiple cognitive and behavioral ED domains (e.g., weight, shape, food, eating, exercise, etc.) and no studies have sought to investigate the combination of general *and* ED specific cognitive-behavioral flexibility. The current, twofold study aimed at construing and validating a comprehensive measure that would allow for the assessment of both general and ED specific cognitive-behavioral flexibility. The aim of Study 1 was to uncover the underlying structure of this new measure through principal component analysis (PCA), and to establish its psychometric properties in terms of internal consistency and convergent validity. Analyses were performed using data collected from individuals with self-reported EDs (i.e., in a clinical sample). The aim of study 2 was to test the three factor model found in Study 1 using confirmatory factor analysis (CFA), and to investigate the measure’s accuracy in distinguishing flexibility scores in individuals with, and without EDs.

## Study 1: Development and Validation of the EDFLIX in a Clinical Sample

### Materials and Methods

#### Questionnaire Framework and Design

A 90-item draft questionnaire focusing on general, and ED specific flexibility was created by a group of researchers and health care professionals at the Regional Department for Eating Disorders (RASP), Oslo University Hospital, Ullevål HF. The pool of items was generated by modifying existing items sourced from questionnaires focusing flexibility, executive function, perfectionism, obsessive compulsive traits, body image and EDs, as well as based on clinical expertise. The 90-item draft questionnaire was then discussed in two separate focus groups, one consisting of five licensed clinical professionals and three Ph.D. level researchers, and the other consisting of three patients recruited from the inpatient ward at RASP. Feedback from the two focus groups resulted in an iterated, 51-item pilot scale consisting of 24 items focusing on general flexibility, and 27 items focusing on ED specific flexibility. This version was worded in Norwegian.

#### Sample and Recruitment

Participants were recruited nationwide through a number of channels. Several ED clinics across Norway (see section “Acknowledgments”) helped spread information about the study by placing pamphlets in their waiting rooms, or posting them on information boards. In addition, the two largest ED user organizations in Norway, Rådgivning om Spiseforstyrrelser (ROS) and Spiseforstyrrelsesforeningen (SPISFO) posted information about the study on their Facebook profile pages encouraging users to participate. Recruitment was also conducted through the Facebook profile page of the RASP research team. All individuals who agreed to participate were asked to fill out a set of questionnaires online (via Nettskjema, a tool for designing and conducting online surveys operated by Oslo University). Eating disorder diagnoses were based on self-report. All participants who responded “*Yes*” to the survey question “*Do you have an eating disorder*?” were included in the Study 1 sample. Participants were prompted to choose one of the following ED diagnoses “*Anorexia Nervosa*,” *Bulimia Nervosa*,” *Binge Eating Disorder*,” or “*Other.*” Both males and females were invited to participate as long as they were at, or above the age of 16. Informed consent was signed online, and all data was collected anonymously. The study was approved by the Norwegian Regional Committee for Medical and Health Research Ethics (Reference No. 2016/863) and the Norwegian Data Protection Authority (Personvern) at Oslo University Hospital.

#### Assessment

##### The Eating Disorder Flexibility Index (EDFLIX)

Fifty one-item pilot scale assessed flexibility the previous 4 weeks (28 days). The items in this scale were selected to capture features of general flexibility (e.g., *“If I have to, it’s easy for me to change my plans”*) and ED specific flexibility (e.g., “*I prefer eating the same foods as I usually do*”). Respondents rated the degree to which they agreed or disagreed with the 51 statements using a six-point Likert scale ranging from 1 (*“Strongly disagree”*) to 6 (*“Strongly agree”*). A number of items should be reversed when scoring (see Table 1 and Supplementary Materials). Higher scores indicate higher levels of general and ED specific flexibility.

**Table 1:** Factor analysis and factor loadings for the 36-item Eating Disorder Flexibility Index (EDFLIX) Questionnaire.

EDFLIX item	Factor 1	Factor 2	Factor 3
(5) I find it difficult to get used to new situations.^r^	0.76		
(4) I think I handle changes well.	-0.72		
(30) I am usually quite flexible.	-0.72		
(16) It’s easy for me to adapt to changes in my environment (e.g., a new workplace/school/home/new friends/colleagues, etc.)	-0.71		
(6) Sudden changes make me distressed.^r^	0.70		
(32) I am a flexible person.	-0.69		
(31) When things don’t go according to plan, I am able to consider alternative solutions.	-0.68		
(21) I am open to new ways of doing things.	-0.65		
(17) I get anxious or distressed if others interfere with my plans.^r^	0.62		
(14) It bothers me when things don’t go exactly as planned.^r^	0.61		
(36) There are usually a number of different ways of doing things.	-0.59		
(13) When I am stuck on a task, I am unable to come up with new solutions.^r^	0.58		
(11) I find it easy to do several things at once.	-0.56		
(23) If I have to, it’s easy for me to change my plans.	-0.53		
(25) I find it difficult when something unexpected happens.^r^	0.52		
(35) I get angry or upset when people don’t do things my way.^r^	0.52		
(27) I find it difficult to consider a situation from several perspectives.^r^	0.49		
(33) Even when I have decided to work out, it’s easy for me not to do it.		-0.77	
(3) I have no specific eating rules that I have to follow.		-0.75	
(1) Even when I have decided what to eat, it is easy for me to eat something else.		-0.67	
(9) I have to exercise a certain number of minutes/hours each day/week.^r^		0.64	
(26) I need my meals to be predictable (time, food, content).^r^		0.63	
(10) I feel I have to follow a set exercise routine.^r^		0.60	
(20) It doesn’t really matter where I eat (e.g., in the kitchen, in the living room, in front of the TV).		-0.58	
(15) From day to day, I am ok with eating my regular meals (breakfast, lunch and dinner at different times.		-0.58	
(19) It is not important to me that a meal lasts a certain amount of time.		-0.56	
(34) I prefer eating the same foods as I usually do.^r^		0.54	
(22) I often try new types of food.		-0.52	
(2) Before I can eat, the food has to be plated in a certain way.^r^		0.49	
(24) I have no problem with other people preparing or cooking my food.		-0.43	
(12) When I start thinking about my weight, I find it difficult to think about anything else.^r^			0.86
(8) If I start thinking about my body, I find it difficult to think about anything else.^r^			0.84
(28) If I think about food, body shape and weight, it is almost impossible for me to stop.^r^			0.84
(18) If I start feeling fat, I cannot think of anything else.^r^			0.77
(29) I get distressed if I gain weight, no matter what I weigh.^r^			0.65
(7) If I am unable to weigh myself when I have planned to, I get distressed/anxious.^r^			0.47


*Only loadings > 0.4 are presented. Three factors with 17 (Factor 1), 13 (Factor 2), and 6 (Factor 3) items respectively were generated. Factor 1 was labeled general flexibility (EDFLIX-GF), Factor 2 was labeled food and exercise flexibility (EDFLIX-FoEx), and Factor 3 was labeled weight and shape flexibility (EDFLIX-WeSh). ^*r*^ = reversed items. Items 1–36 are English translations of the original, Norwegian EDFLIX version, and as such, the English version requires validation.*

##### The Behavior Rating Inventory of Executive Function^^®^^ Adult version (BRIEF-A) (Roth et al., 2005)

We used the *Shift* subscale from the BRIEF-A self-report questionnaire. The BRIEF-A is a standardized questionnaire assessing executive functioning in daily life settings. Responses are scored on a three-point Likert scale: “*never*” (0), “*sometimes*” (1), and “*always*” (2). The S*hift* subscale contains six items measuring the respondent’s ability to move freely from one situation, activity or aspect of a problem to another, as the circumstances demand (Gioia et al., 2000). The items measure both behavioral shift (e.g., “*I get disturbed by unexpected changes in my daily routine*”) and cognitive shift (“*I have trouble thinking of a different way to solve a problem when stuck*”). Higher scores indicate lower levels of flexibility. The psychometric properties of the Norwegian version, used in this study, has proved acceptable, and support the use of American norm data and the reliability of clinical index scores (Dahlgren et al., 2014).

##### The Eating Disorder Diagnostic Scale (EDDS) DSM-5 version

The Eating Disorder Diagnostic Scale (EDDS) is a brief, 23-item self-report questionnaire structured around the DSM-5 criteria for AN, BN, BED, purging disorder and night eating syndrome. The scales consist of a combination of Likert scores, dichotomous scores, frequency scores and open-ended items regarding weight, height and gender. An overall ED symptom composite score can be computed by summing up raw scores across items 1–17. The original 22-item scale based on the DSM-IV criteria has good psychometric properties (Stice et al., 2000) and an overall symptom cut-off score of 16.5 has shown to accurately distinguish between patients with EDs and healthy controls (HCs) (Krabbenborg et al., 2012). The EDDS DSM-5 was translated to Norwegian for the purpose of this study, but has not yet been validated.

##### Sociodemographic information and eating disorder history

This questionnaire included items on self-reported gender, age, educational level, employment status, height, weight. It also included the following six items regarding ED history (response options in brackets): (1) “*Do you have an eating disorder”* (Yes, No, I don’t know), (2) “*How old were you when you developed an eating disorder*?” (Empty response field), (3) “*What diagnosis/type of eating disorder do you have?”* (Anorexia Nervosa, Bulimia Nervosa, Binge Eating Disorder, Other, I don’t know, I don’t have an eating disorder), (4) “*In what city/town is your treatment facility located*” (Empty response field), (5) “*What kind of treatment do you receive*?” (Inpatient treatment, Day treatment, Outpatient treatment, Other, I am not in treatment for an eating disorder), (6) “*How long have you been in treatment for an eating disorder?”* (Less than 1, 1–3, 4–6, 7–10, more than 10 years, I haven’t been in treatment for an eating disorder).

#### Statistical Analyses

All statistical analyses were carried out using IBM SPSS Statistics Version 23. An *a priori* power analysis was not performed; rather, the sample size was based on the widely accepted number of people (N) to the number of measured variables (p) ratio (the N:p ratio) suggesting a minimum of 10 cases (i.e., people) per item (Nunnally and Bernstein, 1967; Wang and Wang, 2012). PCA with oblique rotation (correlations among factors were expected) was conducted to examine the 51-item Eating Disorder Flexibility Index (EDFLIX) questionnaire. The suitability of factor analysis was confirmed by a Kaiser-Meyer-Olkin value of 0.90, exceeding the recommended value of 0.6 (Tabachnick and Fidell, 2007). The Bartlett’s Test of Sphericity (Bartlett, 1954) reached statistical significance (*p* < 0.001), further supporting the factorability of the correlation matrix. The criterion for the number of factors to be rotated was eigenvalues greater than 1, and items with factor loadings lower than 0.4 were excluded. Cronbach’s α coefficient was calculated to determine the internal consistency of the EDFLIX and BRIEF scales (0.8 > α ≥ 0.7 = Acceptable, 0.9 > α ≥ 0.8 = Good, α ≥ 0.9 = Excellent). Construct validity was investigated using Pearson’s correlations applying the following *r* effect sizes: small (0.10), medium (0.30), and large (0.50). Due to a technical glitch, two of the EDDS items were mistakenly omitted in the online questionnaire; item 15 “*Fasted (skipped at least 2 meals in a row)*” and item 21 “*What is your highest weight at your current height*?” After conferring with the creator of the measure, the decision was made to calculate the EDDS symptom composite score despite missing one of the items (item 15), but not to examine diagnoses. The EDDS symptom composite scores should be interpreted with this limitation in mind. Taking into account the diagnostic variability in the clinical sample, it was important to include items targeting ED specific flexibility across diagnostic categories when establishing construct validity. Two individual EDDS items hypothesized to be associated with ED specific flexibility were therefore selected, one relevant to AN and BN (Item 3: “*Has your weight or shape influenced how you judge yourself as a person?”)* and the other one relevant to both BN and BED (Item 6: “*How many times per month on average over the past 3 months have you eaten an unusually large amount of food and experienced a loss of control?*”). A mean compensatory behavior subscale score relevant to all three ED diagnoses (i.e., AN, BN, and BED) was calculated using EDDS item 13 (“Made yourself vomit?”), item 14 (“Used laxatives or diuretics”) and item 16 (“Engaged in more intense exercise specifically to counteract the effects of over eating”), and used in the correlation analyses (see Table 3). We hypothesized that the BRIEF *Shift* subscale and the EDFLIX would be able to capture theoretically related constructs, and therefore chose to include this subscale with the purpose of establishing convergent validity. Based on existing research supporting a negative association between weight and flexibility, BMI was also included. In addition, as emerging evidence suggest that individuals with BN and BED also struggle with flexibility, two additional items tapping into BN and BED specific symptoms were included. These were EDDS item 6 (probing for binge eating), and the EDDS mean compensatory behavior subscale (see above) targeting vomiting, laxative misuse, and excessive exercise.

### Results

A total of 258 participants (255 females) completed the online questionnaire, 44.2% (*N* = 114) of which reported suffering from AN, 23.6% (*N* = 61) from BN and 12.4% (*N* = 32) from BED. 14.3% (*N* = 37) reported “Other” and 5.4% (*N* = 14) reported “I don’t know” on the question regarding ED diagnosis. The latter two groups were excluded from the subsequent analyses, resulting in a final N of 207 participants (204 females and 3 males). In this, selected, sample, mean age was 29.6 years (*SD* = 10.4, range: 16–59). Mean BMI (kg/m^2^) in the three diagnostic groups were 18.1 (*SD* = 2.4, range: 12.3–24.3) for AN, 22.3 (*SD* = 4.6, range: 15.8–35.8) for BN and 34.9 (*SD* = 8.8, range: 19.6–59.6) for BED. The majority of participants with AN and BN reported receiving either inpatient (AN: 17.5%, BN: 1.6%) or outpatient (AN: 44.7%, BN: 60.7%) treatment for their ED. For participants with BED, numbers were lower with 34.4% reported being in outpatient treatment, and 3.1% in inpatient treatment. 46.9% of participants with BED reported not receiving treatment at all. Corresponding numbers for AN and BN were 21.1 and 29.5% respectively. A total of 31.4% of the sample reported being students whereas 22.2% reported being full time employed. Nearly a third of the sample (32.3%) reported being either on sick leave (16.4%) or being incapacitated (15.9%), with the BED group reporting the highest rates (sick leave 12.5%, incapacitated 25.0%). Corresponding numbers for the other two groups were 20.2 and 14.0% for AN, and 11.5 and 14.8% for the BN group.

#### Factor Analysis

The 51-item pilot scale revealed the presence of 12 factors with eigenvalues above 1, together explaining 65.8% of the variance. An inspection of the scree plot revealed a clear break after the fourth factor, which was chosen for further investigation. The analysis revealed the presence of a fairly straight forward structure with all four factors showing a number of strong loadings, and with the vast majority of items loading on one component only. However, seven cross loadings (none of which loaded on more than two factors) were identified indicating relevance to more than one factor. A fixed, three- and five-factor extraction was therefore performed to investigate the possibility of achieving a more optimal solution. The five-factor solution generated additional cross loadings (and showed less conceptual sense), and was discarded. A three-factor solution revealed the presence of a much simpler structure (Table 1), with only three items loading on multiple factors. Cross loading items were assigned to the factor to which they had the highest loading. Finally, five items presenting loading values < 0.4, and four “twin-items” (i.e., items with a close, conceptual resemblance) were removed, resulting in a 36-item final version of the EDFLIX questionnaire. The three-component solution explained a total of 40.7% of the variance, with Factors 1, 2, and 3 contributing with 26.1, 8.4, and 6.2%, respectively. Factor 1 seemed to capture general flexibility, whereas Factor 2 appeared to capture ED specific flexibility related to food and exercise, and Factor 3; ED specific flexibility concerning body shape and weight. Factor 1 was labeled General Flexibility (EDFLIX-GF), Factor 2 was labeled Food and Exercise Flexibility (EDFLIX-FoEx) and Factor 3 was labeled Weight and Shape Flexibility (EDFLIX-WeSh). The final 36-item version was translated into English by a group of researchers and clinicians at RASP, two of which were native English speakers, and one fluent in both Norwegian and English. The EDFLIX questionnaire and scoring instructions are available as Supplementary Materials.

#### EDFLIX, BRIEF-A, EDDS Scales, and Internal Consistency

Descriptive data (including means and standard deviations) for participants in Study 1 (and Study 2) are presented in Table 2. Reported data includes age, BMI, DOI, EDFLIX scale scores, the BRIEF-A shift subscale score and the EDDS composite score. Reliability analyses yielded excellent internal consistency for the EDFLIX total score (Cronbach’s α = 0.91), good internal consistency for the EDFLIX-GF subscale (Cronbach’s α = 0.88) and the two ED flexibility subscales, EDFLIX-FoEx (Cronbach’s α = 0.87) and EDFLIX-WeSh (Cronbach’s α = 0.86). Cronbach’s alpha for the BRIEF-A Shift subscale was acceptable (α = 0.76). Internal consistency was not calculated for the EDDS symptom composite score due to the missing item (item 15). On item-level, all EDFLIX scale response options ranging from 1 (“Strongly disagree”) to 6 (“Strongly agree”) were utilized by respondents, indicating that the chosen rating scale adequately captures the range of cognitions and behaviors regarding flexibility in the clinical sample. Correlations between EDFLIX total and subscales were significant at the *p* < 0.01 level, with medium to large effect sizes (range = 0.35 to 0.84).

**Table 2:** Descriptive data and *post hoc* comparisons for participants with EDs (Study 1) and HCs (Study 2).

	Study 1 (*n* = 207)								Study 2 (*n* = 288)		*Post hoc* Tukey’s HSD
			
	ED total sample (*n* = 207)		AN (*n* = 114) (A)		BN (*n* = 61) (B)		BED (*n* = 32) (C)		HC total sample (D)		A, B, C, and D group comparisons [Cohen’s *d*]
						
	M(*SD*)	Range	M(*SD*)	Range	M(*SD*)	Range	M(*SD*)	Range	M(*SD*)	Range	
Age (years)	29.6 (10.4)	16–59	27.0 (10.0)	16–56	29.7 (8.4)	17–56	38.4 (11.0)	19–59	39.4 (8.8)	16–63	AD^[1.1]^, AD^[1.3]^, BC^[0.9]^, BD^[1.1]^
BMI (kg/m^2^)	22.0 (7.5)	12.3–58.6	18.1 (2.4)	12.3–24.3	22.3 (4.6)	15.8–35.8	34.9 (8.8)	19.6–58.6	25.0 (4.5)	16.7–46.4	AB^[1.1]^, AC^[2.6]^, AD^[1.9]^, BC^[1.8]^, BD^[0.6]^
DOI (years)	13.7 (10.3)	1–53	11.1 (9.1)	1–36	13.6 (8.6)	1–39	22.5 (12.8)	2–53	–	–	AC^[1.0]^, BC^[0.8]^
EDFLIX *t*otal	104.2 (26.0)	36–183	95.4 (23.4)	36–165	111.0 (23.6)	61–180	122.4 (26.1)	68–183	176.5 (19.9)	105–216	AB^[0.7]^, AC^[1.1]^, AD^[3.7]^, BD^[3.0]^, CD^[2.3]^
*EDFLIX-GF*	52.5 (14.0)	17–98	50.0 (13.8)	17–92	54.9 (13.5)	21–92	56.7 (14.1)	19–98	81.1 (11.5)	31–102	AC^[0.5]∗^, AD^[2.5]^, BD^[2.1]^, CD^[1.9]^
*EDFLIX-FoEx*	39.2 (12.7)	13–76	33.8 (10.5)	13–67	43.7 (11.2)	25–70	49.7 (12.7)	28–76	64.7 (8.7)	37–78	AB^[0.9]^, AC^[1.4]^, AD^[3.2]^, BC^[0.5]∗^, BD^[2.1]^, CD^[1.4]^
*EDFLIX-WeSh*	12.5 (5.8)	6–32	11.6 (5.2)	6–27	12.4 (5.9)	6–31	16.0 (6.2)	6–32	30.4 (4.8)	12–36	AC^[0.8]^, AD^[3.8]^, BC^[0.6]^, BD^[3.3]^, CD^[2.6]^
BRIEF-A shift	12.9 (2.5)	2–18	13.2 (2.6)	2–18	12.8 (2.2)	7–18	12.7 (2.6)	7–17	9.3 (2.1)	6–17	AD^[1.7]^, BC^[1.6]^, CD^[1.4]^
EDDS SCS	39.7 (17.8)	12–81	32.9 (17.7)	12–81	49.5 (14.3)	16–75	44.9 (13.5)	25–81	12.1 (7.3)	3–47	AB^[1.1]^, AC^[0.8]^, AD^[1.6]^, BD^[3.3]^, CD^[3.0]^


*ED, eating disorder; AN, anorexia nervosa; BN, bulimia nervosa; BED, binge eating disorder; HC, healthy control; BMI, body mass index; DOI, duration of illness; EDFLIX-GF, general flexibility; EDFLIX-FoEx, food and exercise flexibility; EDFLIX-WeSh, weight and shape flexibility; BRIEF-A Shift, the Behavior Rating Inventory of Executive Function – Adult Version Shift subscale; EDDS SCS, Eating Disorder Diagnostic Scale Symptom Composite Score. Analyses of variance (ANOVA) are performed for group A, B, C, and D. For ED and HC total sample comparisons, see independent sample *t*-test in text. Results from *post hoc* Tukey tests have used *p* < 0.01 unless marked with an ^∗^*p* < 0.05. Cohen’s *d* effect sizes are reported in superscript brackets using the following denominations *d* = 0.2–0.5 (small), *d* = 0.5–0.8, (medium), *d* = ≥ 0.8 (large).*

#### Construct Validity: Scale, Demographic and Anthropometric Variable Correlations

Correlational analyses (Table 3) showed medium to large (range = 0.30 to 0.82) correlations between the EDFLIX total and subscale scores, and the BRIEF *Shift* score indicating good convergent validity. The vast majority of these were significant at the *p* < 0.01 level. This was true for the sample as a whole, as well as for the three diagnostic subgroups. In general, associations between age and flexibility (as measured by the EDFLIX) were stronger in the BN and BED groups where younger patients displayed higher levels of general-, as well as food and exercise related flexibility. With the exception of shape and weight flexibility, shape and weight concerns (as measured by EDDS item 3) were negatively associated with total and subscale scores of flexibility in the AN and BN group, but not in BED. Medium and large negative correlations (range: 0.32 to -0.70) significant at the *p* < 0.01 and *p* < 0.05 level were found between the EDDS compensatory behavior subscale and the EDFLIX total and subscale scores in the BN and BED group, but not in the AN group. With the exception of one significant association between BMI and food and exercise flexibility in the BN group (*r* = 0.31, *p* < 0.05), BMI was not correlated with flexibility as assessed using the EDFLIX.

**Table 3:** Correlations between EDFLIX, BRIEF-A *Shift*, EDDS, and BMI in individuals with EDs (*n* = 207).

	EDFLIX total	EDFLIX-GF	EDFLIX-FoEx	EDFLIX-WeSh
**ED total (*n* = 207)**				
BMI (kg/m^2^)	0.31^**^	0.10	0.43^**^	0.21^**^
BRIEF-A *shift*	-0.62^**^	-0.68^**^	-0.37^**^	-0.33^**^
EDDS *symptom composite score*	-0.08	-0.10	0.05	-0.21^**^
*Shape and weight (Item 3)*	-0.34^**^	-0.24^**^	-0.23^**^	-0.43^**^
*Binge eating (Item 6)*	0.24^**^	0.11	0.35^**^	0.05
*Compensatory behaviors (Items 13,14,16)*	-0.25^**^	-0.19^**^	-0.18^**^	-0.28^**^
**AN (*n* = 114)**				
BMI (kg/m^2^)	0.10	0.02	0.14	0.10
BRIEF-A *shift*	-0.57^**^	-0.61^**^	-0.34^**^	-0.30^**^
EDDS *symptom composite score*	-0.20^*^	-0.21^*^	-0.05	-0.25^**^
*Shape and weight (Item 3)*	-0.43^**^	-0.29^**^	-0.36^**^	-0.46^**^
*Binge eating (Item 6)*	0.02	-0.04	0.11	-0.04
*Compensatory behaviors (Items 13,14,16)*	-0.22^*^	-0.23^*^	-0.09	-0.23^*^
**BN (*n* = 61)**				
BMI (kg/m^2^)	0.09	-0.08	0.31^*^	-0.04
BRIEF-A *shift*	-0.73^**^	-0.76^**^	-0.45^**^	-0.31^*^
EDDS *symptom composite score*	-0.31^*^	-0.17	-0.25	-0.35^**^
*Shape and weight (Item 3)*	-0.39^**^	-0.24	-0.25	-0.52^**^
*Binge eating (Item 6)*	-0.01	-0.02	0.06	-0.13
*Compensatory behaviors (Items 13,14,16)*	-0.40^**^	-0.19	-0.45^**^	-0.32^*^
**BED (*n* = 32)**				
BMI (kg/m^2^)	-0.04	-0.10	0.06	-0.03
BRIEF-A *shift*	-0.74^**^	-0.82^**^	-0.37^*^	-0.49^**^
EDDS *symptom composite score*	-0.52^**^	-0.28	-0.54^**^	-0.45^**^
*Shape and weight (Item 3)*	-0.18	-0.12	-0.05	-0.38^*^
*Binge eating (Item 6)*	0.03	0.10	0.03	-0.15
*Compensatory behaviors (Items 13,14,16)*	-0.64^**^	-0.39^*^	-0.70^**^	-0.40^*^


*BMI, body mass index; ED, eating disorder; AN, anorexia nervosa; BN, bulimia nervosa; BED, binge eating disorder; EDFLIX-GF, general flexibility (items 1–17); EDFLIX-FoEx, food and exercise flexibility (items 18–30); EDFLIX-WeSh, weight and shape flexibility (items 31–36). BRIEF *Shift*, the Behavior Rating Inventory of Executive Function *Shift* subscale. ^∗∗^Pearson’s correlation is significant at the 0.01 level, ^∗^Pearson’s correlation is significant at the 0.05 level; Pearson’s *r* effect sizes = small (0.10), medium (0.30), and large (0.50). Bold figures in the table mark *r* ≥ 0.30 (i.e., effect sizes medium and above).*

## Study 2: Validation of the EDFLIX in a Community Sample

### Materials and Methods

#### Sample, Recruitment, Procedure, and Assessment

Community sample participants, hereafter referred to as HCs, were recruited through the same channels as the clinical sample, and assessed using identical assessment measures. This sample consisted of those who responded “No” to question “*Do you have an eating disorder”* in the online questionnaire section *Sociodemographic information and eating disorder history*. The sample size in Study 2 was based on the N:p ratio-based sample size in Study 1, and the aim was to obtain two samples equal in size. Descriptive data is presented in Table 2.

#### Statistical Analyses

The three factor model found in Study 1 was tested using CFA with the maximum likelihood discrepancy method. CFA was conducted using IBM^^®^^ SPSS^^®^^ Amos^TM^ 20.0. Modification indexes were examined to determine the co-variance between errors that improved the model fit. Cronbach’s alpha was computed for internal consistency. Fit indexes indicate a good fit when: χ^2^ (CMIN) is non-significant (*p* > 0.05); HOELTER 0.05 > 200; root mean square error of approximation (RMSEA) < 0.05 and < 0.08 for acceptable fit; PCLOSE > 0.05; comparative fit index (CFI) > 0.95 for good fit and > 0.90 for acceptable fit; normed fit index (NFI) > 0.95; NNFI (TLI) > 0.90 (Hair et al., 2010; Awang, 2012). Additionally, receiver operating characteristic (ROC) curves were performed using MedCalc Statistical Software version 15.11.4. and areas under the ROC curve (AUCs) were compared. Guidelines for AUC analyses define comparisons as: non-informative (AUC = 0.50), less accurate (0.50 > AUC ≤ 0.70), moderately accurate (0.70 > AUC ≤ 0.90), highly accurate (0.90 > AUC < 1), and perfect (AUC = 1) (Swets, 1988). Youden Index values were used to determine the optimal cutoff score by calculating the maximal combination of sensitivity and specificity of the measure. Youden Index values have been shown to yield lower misclassification rates than other commonly used methods to determine cutoff (Perkins and Schisterman, 2006). Construct validity was investigated using Pearson’s correlation coefficient, applying the following *r* effect sizes: small (0.10), medium (0.30), and large (0.50). BRIEF-A Shift, and EDDS composite and scale scores were calculated identically in Study 1 and Study 2. Cronbach’s α coefficient was calculated to determine the internal consistency of the EDFLIX and BRIEF scales (0.8 > α ≥ 0.7 = Acceptable, 0.9 > α ≥ 0.8 = Good, α ≥ 0.9 = Excellent). An independent sample *t*-test using *p* < 0.01 was performed to investigate differences between individuals with EDs (including all three diagnostic groups) and HCs. Due to the moderate to high correlations between the EDFLIX total and subscale scores, multiple one-way analyses of variance (ANOVA) were performed to investigate group differences in general- and ED specific flexibility. *Post hoc* Tukey’s HSD tests were chosen to account for multiple comparisons (Maxwell and Delaney, 2004), and were performed to identify *where* the difference amongst group were occurring (see Table 2). Effect sizes were calculated using Cohen’s *d* and interpreted in line with Cohen’s classifications (Cohen, 1988) 0.3 = small, 0.5 = medium, and 0.8 = large.

### Results

A total of 288 HCs (286 females) completed the online questionnaire, with ages ranging from 16 to 63 (Mean = 39.4, *SD* = 8.8). A total of 88% of the sample reported being either full time (84.2%) or part time employed (3.8%), whereas 8.2% were students. Five participants (1.7%) reported being on sick leave, and 1.0% reported being incapacitated. The majority (59.5%) had four or more years of university or college education.

#### Confirmatory Factor Analysis

**FIGURE 1:**
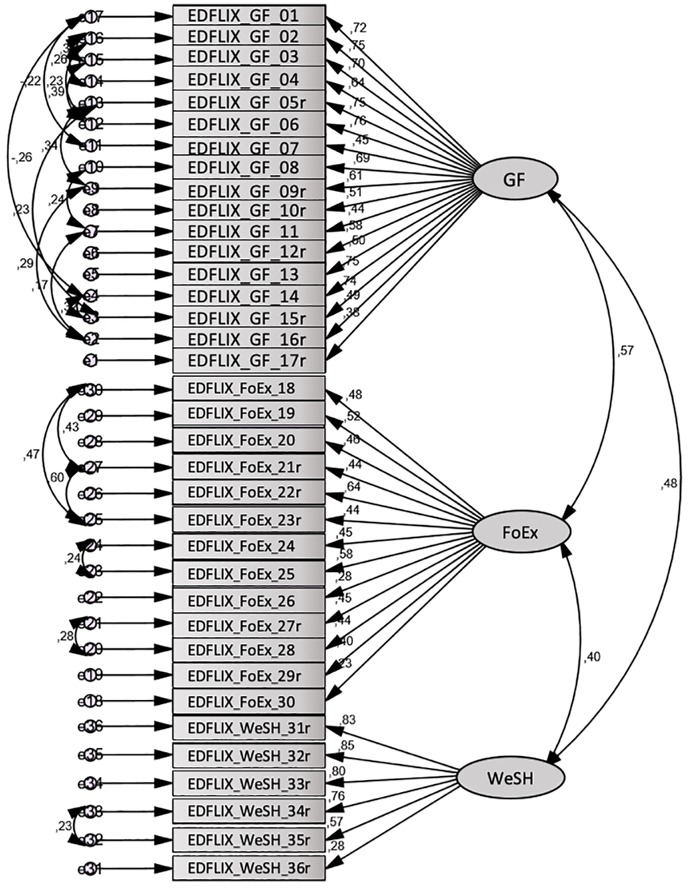
Confirmatory factor analysis of the three-factor EDFLIX model.

The fit indices for the tested model (see Figure 1) were: CMIN = 1013.79; DF = 537; *p* = 0.000; CMIN/df = 1.77; HOELTER 0.05 = 179; RMSEA = 0.051; PCLOSE = 0.293; CFI = 0.902; NFI = 0.792; TLI = 0.886. Given only one index for both the absolute fit indices (CMIN and RMSEA) and the incremental fit indices (CFI, TIL, and NFI) needs to fall within the acceptable range (Holmes-Smith and Coote, 2006; Hair et al., 2010), these results suggest the three-factor structure had acceptable fit in the community sample.

#### EDFLIX Scales and Internal Consistency

Descriptive information regarding EDFLIX total and subscale scores in the community sample are presented in Table 2. Correlations between EDFLIX total and subscales were significant at the *p* < 0.01 level, with medium to large effect sizes (range = 0.37 to 0.87). The reliability analyses yielded excellent internal consistency for the EDFLIX total (Cronbach’s α = 0.91) and EDFLIX-GF (Cronbach’s α = 0.91) subscale in the community sample. The EDFLIX-WeSh and EDFLIX-FoEx subscales possessed good (Cronbach’s α = 0.83) and acceptable (Cronbach’s α = 0.76) internal consistency. Similar to the internal consistency in the clinical sample, Cronbach’s alpha for the BRIEF-A Shift subscale in the community sample was acceptable (α = 0.74).

#### Discriminant Validity

**FIGURE 2:**
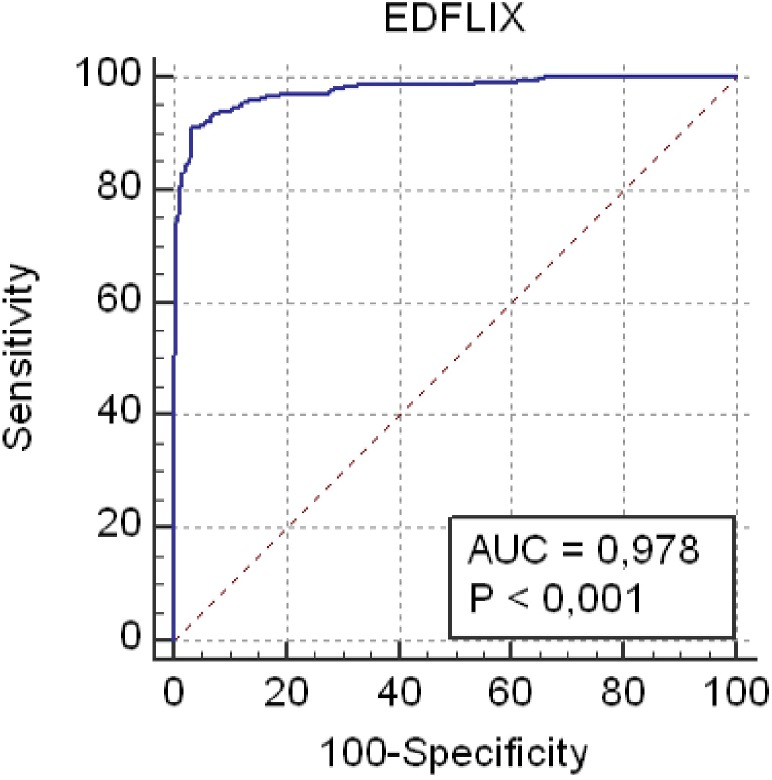
The receiver operating characteristic (ROC) curve.

The mean EDFLIX score for the total sample (*N* = 495) was 146.2 (*SD* = 42.3). For individuals with a current, self-reported diagnosis of an ED (*N* = 207), the average EDFLIX total score was 104.2 (*SD* = 26.0), whereas the mean for HCs (*N* = 288) was 176.5 (*SD* = 19.9). As shown in Figure 2, the ROC analysis demonstrated excellent accuracy distinguishing EDFLIX total scores from individuals with EDs compared to scores from HCs. Specifically, there was a 98% probability that a randomly selected ED case would obtain a lower EDFLIX score than a randomly selected control case (AUC 5 0.98; 95% CI: 0.96–0.99; *p* < 0.01). A cutoff score of 136 on the EDFLIX maximized the Youden index (*J* = 0.88), which indicated the most balanced sensitivity and specificity. At the cutoff point, sensitivity was 0.91 (95% CI: 0.87–0.94) and a specificity of 0.97 (95% CI: 0.94–0.98). Using the 136 cut-off score, for HCs, 8 out of 288 (2.7%) scored below the cut-off score and 21 of 207 (10.1%) of individuals with EDs score above the cutoff.

An independent samples *t*-test was conducted to compare scores on EDFLIX, BRIEF-A *Shift* and EDDS for those with (*n* = 207) and without (HCs) (*n* = 288) EDs. Results revealed that individuals with EDs scored significantly lower on the EDFLIX Total scale *t*(493) = 33.6, *p* < 0.001, *d* = 3.1, on the EDFLIX-GF scale *t*(493) = 24.2, *p* < 0.001, *d* = 2.2, as well as on measures of ED specific flexibility; EDFLIX-FoEx *t*(493) = 25.2, *p* < 0.001, *d* = 2.3, and EDFLIX-WeSh *t*(493) = 36.5, *p* < 0.001, *d* = 3.4. Significantly different score were also found on the BRIEF-A shift subscale *t*(493) = 19.4, *p* < 0.001, *d* = 2.3, and EDDS scale scores *t*(493) = 19.4, *p* < 0.001, *d* = 2.3. Age and BMI were significantly lower in the ED sample; *t*(493) = 10.9, *p* < 0.001, *d* = 1.02 and *t*(493) = 5.76, *p* < 0.001, *d* = 1.02, respectively.

The ANOVA analyses revealed that there was a statistically significant main group (AN, BN, BED, and HC) effect on the EDFLIX Total score: *F*(3,491) = 462,7, *p* < 0.001, *d* = 3.4, on the EDFLIX-GE score: *F*(3,491) = 215,8, *p* < 0.001, *d* = 2.3, on the EDFLIX-FoEx score: *F*(3,491) = 308,2, *p* < 0.001, *d* = 2.7, and on the EDFLIX-WeSh score: *F*(3,491) = 490,9, *p* < 0.001, *d* = 3.5 This was also true for the BRIEF-A shift subscale score: *F*(3,491) = 132.7, *p* < 0.001, *d* = 1.8. The ANOVA analyses also showed a significant group effect for age: *F* (3,452) = 54.2, *p* < 0.001, *d* = 1.2, BMI: *F*(3,489) = 131.2, *p* < 0.001, *d* = 1.8 and EDDS symptom scores: *F*(3,491) = 216.7, *p* < 0.001, *d* = 2.3. *Post hoc* Tukey HSD tests (see Table 2) revealed that the four groups (i.e., the three ED groups and the HCs) were significantly different at the *p* < 0.001 level on most comparisons with medium to large effect sizes (range = 0.5 to 3.8).

#### Construct Validity: Scale, Demographic and Anthropometric Variable Correlations

Correlational analyses (Table 4) showed small to large (range = 0.23 to 0.64) correlations significant at the *p* < 0.01 level between the EDFLIX total and subscale scores, and the BRIEF *Shift* score indicating good convergent validity. The analysis revealed that the EDFLIX-WeSh subscale had the strongest, significant association to ED psychopathology as measured by the EDDS symptom composite score (*r* = -0.63), the EDDS item capturing shape and weight concerns (*r* = -0.59) as well as the compensatory behavior composite score (*r* = -0.39). Age and BMI did not correlate significantly with any of the EDFLIX scales.

**Table 4:** Correlations between EDFLIX, BRIEF-A *Shift*, EDDS, and BMI in HCs (*n* = 288).

	EDFLIX total	EDFLIX-GF	EDFLIX-FoEx	EDFLIX-WeSh
BMI (kg/m^2^)	0.07	0.09	0.08	-0.05
BRIEF-A shift	-0.55^**^	-0.64^**^	-0.23^**^	-0.36^**^
EDDS SCS	-0.42^**^	-0.29^**^	-0.24^**^	-0.63^**^
*Shape and weight (Item 3)*	-0.45^**^	-0.35^**^	-0.25^**^	-0.59^**^
*Binge eating (Item 6)*	-0.11	-0.12^*^	0.02	-0.23^**^
*Compensatory behaviors (Items 13,14,16)*	-0.31^**^	-0.20^**^	-0.24^**^	-0.39^**^


*BMI, body mass index; EDFLIX-GF, general flexibility; EDFLIX-FoEx, food and exercise flexibility; EDFLIX-WeSh, weight and shape flexibility. BRIEF-A Shift, the Behavior Rating Inventory of Executive Function *Shift* subscale; EDDS SCS, the Eating Disorder Diagnostic Scale Symptom Composite Score. ^∗∗^Pearson’s correlation is significant at the 0.01 level, ^∗^Pearson’s correlation is significant at the 0.05 level; Pearson’s *r* effect sizes = small (0.1), medium (0.3), and large (0.5). Bold figures mark effect sizes medium and above (*r* ≥ 0.30).*

## Discussion

The 36-item EDFLIX is a comprehensive, statistically sound measure capturing ED specific and general cognitive-behavioral flexibility. Using PCA and CFA, we identified three internally consistent, theoretically and clinically meaningful subscales: *general flexibility* (EDFLIX-GF), *food and exercise flexibility* (EDFLIX-FoEx) and *weight and shape flexibility* (EDFLIX-WeSh). Results revealed strong psychometric properties with excellent internal consistency and good construct validity. The EDFLIX showed excellent accuracy in distinguishing individuals with and without EDs, and results yielded significant differences in general as well as ED specific flexibility in those with, and without EDs. Overall, individuals with EDs did not differ with regards to general flexibility, but significant differences were found on scores of ED specific flexibility, partly supporting trans-diagnostic differences in EDs, and lending support to its utility for classification purposes.

Study 1 validated the EDFLIX in a sample of individuals with EDs. The PCA supported the questionnaire’s ability to capture both general and ED specific facets of cognitive-behavioral flexibility. The EDFLIX subscales can be considered meaningful on both theoretical and clinical levels, taking into account both diagnostic characteristics and clinical presentations of the disorder. The EDFLIX questionnaire showed good internal consistency confirming the suitability of items and scales chosen, and with alpha values similar to those reported by Roberts et al. (2011). On a group level, significant, negative correlations between EDFLIX total and subscale scores and EDDS symptoms indicated lower levels of flexibility in patients with higher ED symptomatology. This is in accordance with previous studies linking poor cognitive flexibility to more severe ED rituals and longer DOI (Roberts et al., 2010), whereas higher flexibility is associated with lower ED psychopathology (Pellizzer et al., 2018). Specifically, correlations showed that higher shape and weight concerns were associated with lower cognitive-behavioral flexibility potentially tapping into rigidity around ritualistic weighing, body checking as well as preoccupation with weight and shape, as shown in the literature (Øverås et al., 2015; Arlt et al., 2016). In addition, the EDFLIX total and subscale scores are associated with previously validated measures of flexibility (BRIEF *Shift*) and ED symptoms (EDDS) indicating that the instrument captures the mechanisms it was intended to measure. In the current study, flexibility was not associated with BMI. This finding is in line with a number of recent studies on cognitive functioning in EDs (Weider et al., 2015; Kjaersdam Telleus et al., 2016; Bentz et al., 2017).

Results from Study 2 revealed good psychometric properties for the EDFLIX in the HC sample, with the CFA confirming the three-factor structure found in Study 1. The ROC curve demonstrated excellent accuracy in distinguishing individuals with EDs from those without EDs, and cut-off score of 136 was found to represent the most balanced sensitivity and specificity. None of the three previously published psychometric studies (Roberts et al., 2011; Sandoz et al., 2013; Duarte et al., 2017) describing ED specific, self-reported flexibility have reported clinical cut-off scores. As such, the EDFLIX is the first assessment instrument to actively separating clinical from non-clinical flexibility scorings. Further, in contrast to the IEQ, the DFlex and the BI-AAQ, the EDFLIX has been developed for, and validated in a transdiagnostic sample, increasing its utility compared to existing self-report measures. Where the DFlex assesses cognitive rigidity and attention to detail, the IEQ targets eating rules and the BI-AAQ assesses body image, the EDFLIX builds on, and expands the knowledge by providing a measure capturing both broad and specific facets of cognitive-behavioral flexibility in EDs. The results from the EFA revealed a clear distinction between general and ED-specific flexibility in individuals with EDs, indicating that difficulties with flexibility stretches beyond that of ED cognitions and behaviors, suggesting individuals with EDs struggle with flexibility on a more global scale than previously documented.

The EDFLIX was designed to capture both cognitive flexibility as well as aspects of behavioral flexibility. Behavioral flexibility is commonly viewed as the ability to change behavior in accordance with changes in the internal or external environment (Brown and Tait, 2014), and related to this is the ability to shift attention between several dimensions of attentional sets. Self-control is also considered an aspect of behavioral flexibility (Coutlee and Huettel, 2012), and traditional tests of self-control often measure the subject’s ability to focus on the most efficient approach to a task, instead of using a more direct or simpler route. Although reversal learning, set-shifting, and self-control are all considered aspects of behavioral flexibility they have distinct differences in terms of the traits measured and their neural substrates (Audet and Lefebvre, 2017). Studies using traditional neuropsychological tests to assess set-shifting rarely use tasks which differentiate between cognitive and behavioral inflexibility (Zastrow et al., 2009; Roberts et al., 2011) even though it has been suggested that impaired flexibility in AN is predominantly caused by a deficit in behavioral response shifting, and not poor cognitive set-shifting (Zastrow et al., 2009). The findings from the current study reveal that participants with AN self-assess both their behaviors and cognitions as inflexible, both in terms of everyday situations as well as with regards to ED specific themes and topics. Future studies might wish to investigate the relationship between EDFLIX scores and traditional neuropsychological tasks to assess whether there are elements of the EDFLIX that more specifically assesses cognitive vs. behavioral flexibility.

Consistently throughout the analyses, HCs showed to be the most flexible, i.e., achieved the highest flexibility scores, followed by individuals with BED, BN and lastly, those with self-reported AN. Participants with BN and BED appeared to self-report flexibility fairly similarly. This is in line with previous studies showing shared behavioral patterns in terms of compulsive overeating for individuals with BN and BED (Hill, 2007; Goldschmidt et al., 2008). Further, all three diagnostic groups scored lower than HCs on general cognitive-behavioral flexibility indicating that rigidity, rule adherence and strict everyday regimens stretch beyond the realm of ED specifics, affecting individuals with EDs on a more global level. These findings are in line with findings from Roberts et al. (2011), but also add to the extant literature by detailing how individuals with BED score significantly higher on general flexibility compared to those with AN, but not compared to those with BN. Duchesne et al. (2010) found cognitive flexibility difficulties in patients with BED compared to obese controls without BED. Dingemans et al. (2019), on the other hand, failed to find differences between HCs and patients with BED on neuropsychological tests of executive function. However, results revealed associations between self-reported difficulties in executive functioning in daily life, ED psychopathology and depressive symptoms. For this reason, the authors propose that “*a self-report questionnaire which evaluates the functional, real-world impact of executive dysfunction expressed in everyday activities seems to be more useful in this population than neuropsychological tests”* (Dingemans et al., 2019, p. 143). The EDFLIX questionnaire may very well be a self-report questionnaire suitable for such assessment purposes. The findings from the current study further revealed that, with the exception of AN and BN, the ED groups were significantly different from each other in terms of flexibility related to weight and shape. The lack of a significant difference between the AN and BN groups on the EDFLIX-WeSh subscale, and the corresponding finding that the BED group significantly differs from both AN and BED on this subscale is not surprising given that overvaluation of weight and shape is a core feature of AN and BN, and critical when assigning diagnoses. Overvaluation of weight and shape is not a diagnostic criterion for BED, which might further help explain these findings. Although all ED groups scored significantly low on flexibility in terms of food and exercise, the largest difference was found when comparing individuals with AN to those without an ED, indicating that AN is most “distinct” when it comes to food and exercise. The smallest difference was between BN and BED, suggesting these two diagnostic groups are, although significantly different, most similar in terms of food and exercise flexibility. These results are in concordance with clinical observations of variations in symptomatic behavior between the three diagnostic groups, with individuals with AN showing more rigidity when it comes to food and exercise regimes and rituals, compared to individuals with BN and BED.

### Strengths and Limitations

The rigorous methodology and the large, diagnostically diverse sample are some of the most important strengths of this study. However, there are also some limitations to note. Although self-reports are widely used to establish ED diagnoses (Dahlgren and Wisting, 2016), and have been used to assess ED psychopathology in studies comparable to the current one (Roberts et al., 2011; Sandoz et al., 2013; Duarte et al., 2017), the reliance of self-reported diagnoses is a major limitation in the current study. Both samples (i.e., individuals with EDs and HCs) are likely to include “false positives” (i.e., people who report having an ED when in fact they do not) as well as “false negatives” (i.e., people who have an ED without reporting or recognizing it). Studies have demonstrated divergent results when using diagnostic interviews and self-reports to assign ED diagnoses, sometimes leading to higher levels of ED psychopathology through self-reports (Passi et al., 2003; Wolk et al., 2005; Dahlgren and Wisting, 2016) and other times lower levels of psychopathology where classification of EDs appears also to be diagnostically specific (Birgegard et al., 2014). Also, as weight and height was based on self-report rather than being objectively measured, BMI rates may be biased. Secondly, we did not control for potential confounding effects of depression and anxiety. Both of these comorbid conditions have shown to impact executive function in EDs (Giel et al., 2012; Abbate-Daga et al., 2015; Ely et al., 2016). Also, because data was collected anonymously, we could not perform test–retest analyses to investigate the stability of the measure. However, by calculating the internal consistency of the questionnaire, we were able to examine the consistency of the questionnaire and omit variables which reduced the inter-correlation between the question variables. Being a self-report measure, the EDFLIX does not quantify cognitive-behavioral flexibility with the same level of specificity as neuropsychological tests. However, one might argue that the EDFLIX might be more clinically useful as it also taps into ED specific cognitions and behaviors. Moreover, clinically observed patterns of cognitive inflexibility are commonly found to be exaggerated with weight loss. As with all self-report measures, a certain level of insight is required for people to accurately rate their tendencies to behave or think in a specific manner. Given the relatively high BMI of the AN group in this study, applying the EDFLIX in inpatient AN settings will help to inform the influence of illness severity on general and ED specific cognitive-behavioral flexibility.

### Suggestions for Future Research

A number of potential research avenues are worth mentioning. Firstly, future studies should validate the measure in formally diagnosed, clinical samples including atypical and sub-threshold EDs, and explore its utility across geographic and cultural bonds. As emerging evidence shows that males and females with EDs share some of the cognitive features (Goddard et al., 2014), and that ED subtypes might present with different neurobiological correlates (Van Autreve et al., 2016), future research should aim to clarify gender and subtype bias in self-reported cognitive and behavioral flexibility. As some recent findings indicate poorer executive functions in BN patients with a prior history of AN (Degortes et al., 2016), another suggestion for future research is to investigate how diagnostic crossover affects self-reported flexibility. In addition, as the vast majority of studies have failed to find performance-based cognitive flexibility deficits in children and adolescents with EDs (Dahlgren and Ro, 2014), we encourage researchers to use the EDFLIX to investigate whether self-reported flexibility may better capture the cognitive and behavioral rigidity often observed in these patients. Finally, future studies should also aim to assess the divergent and predictive validity of the EDFLIX.

As cognitive and behavioral inflexibility make change difficult, it is important to detect patients who struggle with flexibility early on in the treatment process. The EDFIX is easily administered and free of charge, and could, help identifying target areas in need of subsequent attention. The measure could also be used to tailor individual treatment efforts, to supplement existing pre-and post-treatment assessments, or be used as a screening instrument in at-risk populations.

## Data Availability

All datasets generated for this study are included in the manuscript and the Supplementary Files.

## Author Contributions

CD, TH, and KS conceived of the presented idea. CD and JW performed the statistical analyses, which were verified by TH and KS. All authors discussed the results and contributed to the final manuscript.

## Conflict of Interest Statement

The authors declare that the research was conducted in the absence of any commercial or financial relationships that could be construed as a potential conflict of interest.
