# Supplementary material for: General and Eating Disorder Specific Flexibility: Development and Validation of the Eating Disorder Flexibility Index (EDFLIX) Questionnaire
Source: Front Psychol. 2019 Apr 2;10:663. doi: 10.3389/fpsyg.2019.00663 (PMC6454114; doi:10.3389/fpsyg.2019.00663)
Supplement: Supplementary file 1 [file Table_1.pdf]

# The Eating Disorder Flexibility Index (EDFLIX) Questionnaire

Copyright 2019 by Camilla Lindvall Dahlgren, Trine Wiig-Hage and Kristin Stedal

**Instructions:** The statements below are concerned with the past four weeks (28 days) only. Please read each statement and mark a cross in the box which most closely expresses your agreement or disagreement with the statement. Please answer all questions. There are no right or wrong answers.

| | | Strongly disagree | Disagree | Slightly disagree | Slightly agree | Agree | Strongly agree |
|---|---|---|---|---|---|---|---|
| 1. | Even when I have decided what to eat, it is easy for me to eat something else. | ☐ | ☐ | ☐ | ☐ | ☐ | ☐ |
| 2. | Before I can eat, the food has to be plated in a certain way. | ☐ | ☐ | ☐ | ☐ | ☐ | ☐ |
| 3. | I have no specific eating rules that I have to follow. | ☐ | ☐ | ☐ | ☐ | ☐ | ☐ |
| 4. | I think I handle changes well. | ☐ | ☐ | ☐ | ☐ | ☐ | ☐ |
| 5. | I find it difficult to get used to new situations. | ☐ | ☐ | ☐ | ☐ | ☐ | ☐ |
| 6. | Sudden changes make me distressed. | ☐ | ☐ | ☐ | ☐ | ☐ | ☐ |
| 7. | If I am unable to weigh myself when I have planned to, I get distressed/anxious. | ☐ | ☐ | ☐ | ☐ | ☐ | ☐ |
| 8. | If I start thinking about my body, I find it difficult to think about anything else. | ☐ | ☐ | ☐ | ☐ | ☐ | ☐ |
| 9. | I have to exercise a certain number of minutes/ hours each day/week. | ☐ | ☐ | ☐ | ☐ | ☐ | ☐ |
| 10. | I feel I have to follow a set exercise routine. | ☐ | ☐ | ☐ | ☐ | ☐ | ☐ |
| 11. | I find it easy to do several things at once. | ☐ | ☐ | ☐ | ☐ | ☐ | ☐ |
| 12. | When I start thinking about my weight, I find it difficult to think about anything else. | ☐ | ☐ | ☐ | ☐ | ☐ | ☐ |
| 13. | When I am stuck on a task, I am unable to come up with new solutions. | ☐ | ☐ | ☐ | ☐ | ☐ | ☐ |
| 14. | It bothers me when things don't go exactly as planned. | ☐ | ☐ | ☐ | ☐ | ☐ | ☐ |
| 15. | From day to day, I am ok with eating my regular meals (breakfast, lunch and dinner) at different times. | ☐ | ☐ | ☐ | ☐ | ☐ | ☐ |
| 16. | It's easy for me to adapt to changes in my environment (e.g. a new workplace / school/ home/ new friends/colleagues etc.). | ☐ | ☐ | ☐ | ☐ | ☐ | ☐ |
| 17. | I get anxious or distressed if others interfere with my plans. | ☐ | ☐ | ☐ | ☐ | ☐ | ☐ |
| 18. | If I start feeling fat, I cannot think of anything else. | ☐ | ☐ | ☐ | ☐ | ☐ | ☐ |

| | | Strongly disagree | Disagree | Slightly disagree | Slightly agree | Agree | Strongly agree |
|---|---|---|---|---|---|---|---|
| 19. | It is not important to me that a meal lasts a certain amount of time. | ☐ | ☐ | ☐ | ☐ | ☐ | ☐ |
| 20. | It doesn't really matter where I eat (e.g. in the kitchen, in the living room, in front of the TV) | ☐ | ☐ | ☐ | ☐ | ☐ | ☐ |
| 21. | I am open to new ways of doing things. | ☐ | ☐ | ☐ | ☐ | ☐ | ☐ |
| 22. | I often try new types of food. | ☐ | ☐ | ☐ | ☐ | ☐ | ☐ |
| 23. | If I have to, it's easy for me to change my plans. | ☐ | ☐ | ☐ | ☐ | ☐ | ☐ |
| 24. | I have no problem with other people preparing or cooking my food. | ☐ | ☐ | ☐ | ☐ | ☐ | ☐ |
| 25. | I find it difficult when something unexpected happens. | ☐ | ☐ | ☐ | ☐ | ☐ | ☐ |
| 26. | I need my meals to be predictable (e.g. *when* I eat, type of food, food contents etc.). | ☐ | ☐ | ☐ | ☐ | ☐ | ☐ |
| 27. | I find it difficult to consider a situation from several perspectives. | ☐ | ☐ | ☐ | ☐ | ☐ | ☐ |
| 28. | If I think about food, body shape and weight, it is almost impossible for me to stop. | ☐ | ☐ | ☐ | ☐ | ☐ | ☐ |
| 29. | I get distressed if I gain weight, no matter what I weigh. | ☐ | ☐ | ☐ | ☐ | ☐ | ☐ |
| 30. | I am usually quite flexible. | ☐ | ☐ | ☐ | ☐ | ☐ | ☐ |
| 31. | When things don't go according to plan, I am able to consider alternative solutions. | ☐ | ☐ | ☐ | ☐ | ☐ | ☐ |
| 32. | I am a flexible person. | ☐ | ☐ | ☐ | ☐ | ☐ | ☐ |
| 33. | Even when I have decided to work out, it's easy for me not to do it. | ☐ | ☐ | ☐ | ☐ | ☐ | ☐ |
| 34. | I prefer eating the same foods as I usually do. | ☐ | ☐ | ☐ | ☐ | ☐ | ☐ |
| 35. | I get angry or upset when people don't do things my way. | ☐ | ☐ | ☐ | ☐ | ☐ | ☐ |
| 36. | There are usually a number of different ways of doing things. | ☐ | ☐ | ☐ | ☐ | ☐ | ☐ |

Please check that you have answered all 36 questions. Thank you for filling in the questionnaire!

# The Eating Disorder Flexibility Index (EDFLIX) Questionnaire
## Scoring Instructions

A total score (EDFLIX Total Score) and three subscale scores (EDFLIX-GF, EDFLIX-FoEx and EDFLIX-WeSh) can be calculated using these scoring instructions.

The EDFLIX Total score is calculated by summing scores on all 36 items. The subscale scores are calculated by adding together the item scores on each subscale. **Higher scores indicate higher flexibility.**

A number of items should be *reverse scored* (see below). This should be done *before* calculating total and subscale scores.

Score the items as follows:

| | |
|---|---|
| Strongly disagree: | 1 |
| Disagree: | 2 |
| Slightly disagree: | 3 |
| Slightly agree: | 4 |
| Agree: | 5 |
| Strongly agree: | 6 |

*Reverse scored* items should be scored as follows:

| | |
|---|---|
| Strongly disagree: | 6 |
| Disagree: | 5 |
| Slightly disagree: | 4 |
| Slightly agree: | 3 |
| Agree: | 2 |
| Strongly agree: | 1 |

**EDFLIX Total Score**
Sum scores on item 1-36
*Reverse score* the following items: 02, 05, 06, 07, 08, 09, 10, 12, 13, 14, 17, 18, 25, 26, 27, 28, 29, 34, 35

**EDFLIX-GF (General Flexibility)**
Sum scores on items (17 items): 04, 05, 06, 11, 13, 14, 16, 17, 21, 23, 25, 27, 30, 31, 32, 35, 36
*Reverse score* the following items: 05, 06, 13, 14, 17, 25, 27, 35

**EDFLIX-FoEx (Food and Exercise Flexibility)**
Sum scores on items (13 items): 01, 02, 03, 09, 10, 15, 19, 20, 22, 24, 26, 33, 34
*Reverse score* the following items: 02, 09, 10, 26, 34

**EDFLIX-WeSh (Weight and Shape Flexibility)**
Sum scores on items (6 items): 07, 08, 12, 18, 28, 29
*Reverse score* the following items: 07, 08, 12, 18, 28, 29
